# Impacts of Mislabeled ECIG Liquids on Primary Particulate Matter Emissions

**DOI:** 10.3390/toxics14030256

**Published:** 2026-03-13

**Authors:** Sarah E. Fresquez, Vijay Sivaraman, Yogesh Saini, Daniel Walker, Talia Chavis, Eric Soule, Sinan Sousan

**Affiliations:** 1 Department of Public Health, Brody School of Medicine, East Carolina University, Greenville, NC 27834, USA; fresquezs19@ecualumni.ecu.edu; 2 Department of Biological and Biomedical Sciences, College of Health and Sciences, North Carolina Central University, Durham, NC 27707, USA; vijay.sivaraman@nccu.edu; 3 Center for Human Health and the Environment, NC State University, Raleigh, NC 27707, USA; ysaini@ncsu.edu; 4 Department of Population Health and Pathobiology, College of Veterinary Medicine, North Carolina State University, Raleigh, NC 27607, USA; 5 Department of Public Health, College of Health and Human Performance, East Carolina University, Greenville, NC 27858, USA; walkerd22@students.ecu.edu; 6 Department of Public Health Education, College of Health and Sciences, North Carolina Central University, Durham, NC 27707, USA; chavist5@vcu.edu; 7 Department of Health Education and Promotion, East Carolina University, Greenville, NC 27858, USA; soulee18@ecu.edu; 8 North Carolina Agromedicine Institute, Greenville, NC 27858, USA

**Keywords:** electronic cigarettes, particulate matter, vaping emissions, nicotine analysis, PG/VG analysis, ECIG liquid labeling

## Abstract

Electronic cigarette (ECIG) liquids are marketed with labeled nicotine concentrations and propylene glycol (PG) to vegetable glycerin (VG) ratios, yet quality control inconsistencies may alter vaping emissions. We quantified discrepancies between labeled and measured chemical content and evaluated how these differences affect emissions of particulate matter with an aerodynamic diameter of 2.5 µm or smaller (PM_2.5_). Flavor-free liquids (*n* = 20) spanning nicotine labels of 0, 9, 18, and 48 mg/mL and PG content from 0% to 80% were purchased. Nuclear magnetic resonance spectroscopy measured nicotine, PG, and VG. Aerosols were generated using a standardized device in a controlled exposure chamber. PM_2.5_ was measured using a pDR-1500 and SMPS/APS, with gravimetric correction factors calculated. Labeling inaccuracies were widespread: “nicotine-free” liquids contained 0.1 to 0.4 mg/mL nicotine, and labeled nicotine deviated by up to ±30%. PG/VG ratios were frequently incorrect; 70% of samples contained higher VG than labeled, including “100% VG” products with about 10% PG. Higher VG consistently increased PM_2.5_ mass, while nicotine had a minimal effect. The pDR overestimated mass, whereas SMPS/APS underestimated due to volatilization losses. Overall, inaccurate ECIG liquid labeling can alter measured PM_2.5_ emissions under controlled conditions.

## 1. Introduction

Electronic cigarettes (ECIGs) have become popular over the past decade, often marketed as “safe alternatives” compared to normal cigarettes [1]. However, the Centers for Disease Control and Prevention [2] has deemed ECIGs a health risk, primarily due to nicotine, which is a highly addictive substance with well-documented health risks across vulnerable populations [3]. Prenatal nicotine exposure is toxic to developing fetuses and poses serious dangers for pregnant women. Acute exposure can also result in poisoning in both children and adults through ingestion, inhalation, or dermal and ocular absorption of e-liquid [4]. Sadly, ECIGs have become popular among youth in the United States and other countries in the world [5]. Adolescents are especially susceptible due to ongoing brain development, which continues until approximately age 25 [2]. Nicotine use during this critical period can lead to the rapid onset of dependence, disrupt neural pathways involved in attention, learning, mood, and impulse control, and elevate the risk of subsequent substance use disorders [3]. Furthermore, youth who use ECIGs are more likely to initiate cigarette smoking later in life [6,7,8,9,10].

ECIGs are battery-powered devices that use an electric heater (i.e., atomizer) to aerosolize a liquid for inhalation [11]. ECIG liquids typically contain a mixture of nicotine, propylene glycol (PG), vegetable glycerin (VG) and flavorants. ECIG liquids are sold with different nicotine concentrations, as well as salt and non-salt (free-base) nicotine, PG/VG ratios, and various flavorants. Alternatively, they can be mixed by the consumer to create “DIY” (do-it-yourself) liquids [12]. Free-base nicotine is the natural, alkaline chemical form of nicotine, which tends to be bitter and harsh on the respiratory tract when inhaled [13].

In contrast, salt nicotine is created by adding organic acids (such as benzoic acid) to free-base nicotine, converting it into a protonated form that produces a sweeter, smoother, and significantly less harsh vaping experience [13,14]. Additionally, some ECIG liquids are sold with the atomizer or separately, allowing users to vary the liquid content and device components, which impacts the aerosol emitted from an ECIG device [15]. Assessing ECIG health risks requires reliable data on what users are actually inhaling, so the accuracy of this chemical profile/labeling is critical. If the liquid composition is mislabeled, both the estimated toxicity and the physical measurement of the aerosol concentration will be incorrect.

Studies have shown that high PG concentrations result in lower aerosol concentrations compared to higher VG concentrations, due to the higher volatility of PG [11,12,16]. Additionally, ECIG liquids with high concentrations of PG may deliver nicotine more effectively than those with concentrations of VG, similar to free-base nicotine [17]. The ECIG liquid components have combining or different health effects, where studies indicate that the nicotine solvents propylene glycol and glycerol may impact the heart and lungs [18]. Excessive systemic exposure to propylene glycol has been linked to metabolic acidosis, acute kidney injury, and symptoms resembling sepsis. In addition, short-term exposure to Glycol mixtures can cause throat irritation, dry cough, and temporary reductions in lung function, particularly among individuals with higher levels of exposure [18].

ECIG devices emit airborne particles and gases, and specifically PM_2.5_, particulate matter (aerosols) that are 2.5 μm in size and smaller [11]. PM_2.5_ is of concern due to its association with cardiopulmonary diseases, particularly at high concentrations, which are primarily attributed to VG for ECIGs [19,20,21,22,23,24]. Several studies have focused on measuring ECIG-generated airborne contaminants in various settings and have shown that ECIGs generate high concentrations of PM [25,26,27]. Since PM_2.5_ emissions are highly sensitive to solvent composition, inaccuracies in labeled PG/VG ratios can lead to incorrect interpretation of aerosol emission measurements. This makes chemical verification essential when comparing products marketed with different stated nicotine strengths and PG/VG ratios. In addition, the study demonstrated that higher VG liquid concentrations led to higher PM_2.5_ mass concentrations, whereas higher PG liquid concentrations led to lower PM_2.5_ mass concentrations.

Subsequently, Soule, et al. [28] and Sousan, et al. [29] measured the secondhand PM_2.5_ ECIG exposure inside vehicles while participants were vaping and reported concentrations up to 143,504 μg/m^3^. In these studies [11,29], filter correction factors were shown to be crucial for various PM instruments, given their limitations in measuring PM_2.5_ concentrations.

Although previous studies have documented discrepancies between labeled and measured nicotine concentrations in ECIG liquids, less attention has been given to how such chemical mislabeling propagates into aerosol emission measurements and real-time PM_2.5_ quantification. In particular, few studies have examined whether inaccuracies in labeled PG/VG ratios alter expected emission trends, bias instrument response, or affect gravimetric correction factors. Addressing this gap requires pairing chemical verification of liquid composition with controlled aerosol emission measurements. Studies have shown inconsistencies in labeling, contamination, and variations between free-base nicotine and nicotine salts, which raise concerns about the accuracy of quantification and safety [30]. Raymond, et al. [31] found major discrepancies between labeled and actual nicotine concentrations in domestically produced ECIG liquid. Samples labeled 18 mg/mL often varied widely, and most products labeled nicotine-free still contained measurable levels, underscoring risks to consumers and the need for stricter regulation and testing. Research highlights poor quality control of these products and discrepancies in nicotine concentrations. It emphasizes the need for further studies on the long-term health effects of ECIG liquid and its role in smoking cessation [30].

The objective of this study was to chemically verify nicotine concentration and PG/VG ratios in commercially available ECIG liquids and to evaluate how discrepancies between labeled and actual composition influence aerosol emission measurements. Using NMR-based quantification in combination with controlled-chamber experiments, this work directly links chemical mislabeling to PM_2.5_ mass concentrations and to instrument-specific measurement bias. While prior studies have reported nicotine labeling discrepancies and demonstrated higher PM_2.5_ emissions for VG-rich liquids, fewer studies have directly linked chemical mislabeling to the interpretation of aerosol emissions and real-time instrument bias within a single experimental framework. This study integrates (1) NMR-based quantification of nicotine, PG, and VG content, (2) chamber-based PM_2.5_ emission measurements, and (3) gravimetric correction factors to evaluate how mislabeled liquid composition can alter emission trends and measurement accuracy. By linking chemical mislabeling directly to aerosol emission measurements and instrument correction behavior, this study clarifies how inaccuracies in labeled ECIG liquid composition can propagate into errors in emission characterization.

## 2. Materials and Methods

### 2.1. ECIG Device and ECIG Liquid Used

The device used for aerosol generation was a SMOK Novo X (Smoktech, Shenzhen, China) [32] with 0.8 Ω mesh coil pods. A total of 4 devices were used for the experiments. Each device was fully charged for each experiment, and each condition used one new, fully filled pod. The SMOK Novo X was selected due to the widespread popularity of pod-based systems and mesh coils. While the device’s power is adjustable from 1 W to 25 W, it was operated exclusively at 15 W with a standard 0.8 Ω coil. This configuration represents typical user behavior, ensuring stable, consistent aerosol production without overheating the liquid. A fixed 15 W setting was used across all experiments to minimize variability attributable to device power and to isolate the effects of liquid composition. A total of four SMOK Novo X were used, and the batteries were cycled out to allow them to recharge and cool between experiments. Pods were reused after the first trials, and a replacement pod was used for ECIG liquid if the original pod began leaking. Pod fill volume followed the manufacturer’s indicated fill line.

The objective of the study was to select a randomly selected retailer (website) that sells its own unflavored ECIG liquids to minimize additional sources of variability. Therefore, all ECIG liquids were purchased from VapingZone (Electronic Cigarettes 378, Columbiana Drive Ste 4, SC, USA) on 27 October 2023. Twenty flavor-free 60 mL bottles were acquired with free-base nicotine concentrations of 0, 9, 18, and 48 mg/mL, each with PG/VG ratios of 0/100, 30/70, 50/50, 70/30, and 80/20, respectively, as documented in the purchase and shipping records retained by the investigators. Free-base nicotine was selected for this study to isolate the effects of nicotine concentration and PG/VG composition without introducing additional variability from salt-forming acids [33]. A single retailer was used to reduce cross-vendor variability and focus on within-source labeling accuracy across nicotine strengths and stated PG/VG ratios. The nicotine concentrations were designated as N1 through N4, and the PG/VG ratios were designated as R1 through R5. For example, N1R1 represented 0 mg/mL nicotine concentration and a 0/100 PG/VG ratio. The flavorless bottles were selected to focus on the nicotine, PG and VG values, and purchasing these specific concentrations directly from the manufacturer to evaluate variability in the mixtures. The PG and VG ratio densities were determined using the densities of PG (1040 mg mL^−1^) and VG (1260 mg mL^−1^). Three trials were conducted for each liquid, with a new ECIG pod used for the first trial and refilled for trials 2 and 3.

### 2.2. ECIG Liquid Chemical Analysis

A Bruker NEO 600 MHz Nuclear Magnetic Resonance (NMR) spectrometer (Billerica, MA, USA) equipped with a 5 mm BBFO probe was used to perform the chemical analysis of the 20 ECIG liquid bottles. Samples were prepared and analyzed using the methods from Crenshaw, et al. [34] as a starting point. A set amount of 1,2,4,5-tetrachloro-3-nitrobenzene (Santa Cruz Biotechnology, Dallas, TX, USA), ranging from 150 mg to 250 mg, was added to 10 mL of N,N-dimethylformamide-D7 (Cambridge Isotope Laboratories, Tewksbury, MA, USA). A total of 500 microliters of this solvent/standard solution was used for each sample and added to 60 microliters of an ECIG liquid sample. Samples were added to 5 mm NMR tubes for analysis, and the data were collected using the Bruker pulse sequence zg, a five-second acquisition time, a 60-second relaxation delay, 2 dummy scans, and 32 scans. All samples were prepared in new tubes with new caps, each purchased specifically for the study. NMR data were phased, baseline corrected, and peaks integrated using Topspin 4.3.0 (Bruker, Billerica, MA, USA). Quantitation of propylene glycol was done using peaks at 1.083, 3.347, and 3.404 ppm. Quantitation of glycerol was done using peaks at 3.494, 3.564, and 3.6322 ppm. Quantitation of nicotine was done using peaks at 1.679, 1.797, 1.915, 2.117, 2.219, 7.401, 7.7942, 8.510, and 8.573 ppm. The standard peak at 8.401 ppm was used to determine the absolute quantity of the vape e-liquid oil components.

### 2.3. Exposure Chamber

**Figure 1:**
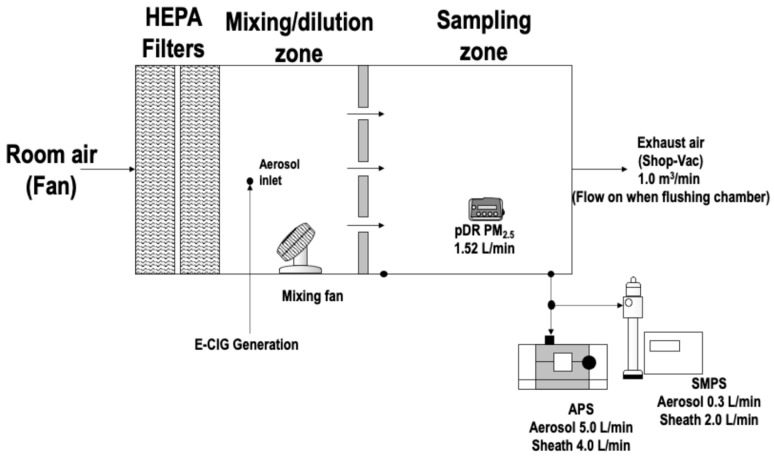
The experimental chamber for measuring ECIG exposure of different mixture ratios.

ECIG emissions were measured within a controlled 0.5 m^3^ exposure chamber consisting of a mixing zone (0.25 m^3^) and a sampling zone (0.25 m^3^), as shown in Figure 1. The chamber volume is adequate to support homogeneous mixing and reproducible instrument comparison under controlled conditions. Concentrations generated in this micro-chamber represent a laboratory emission scenario and are not intended to directly represent realistic indoor bystander exposure levels, which depend on room volume, ventilation, and occupant behavior.

The mixing zone contained two fans that operated during experiments and when flushing the chamber to distribute air to the sampling zone at a flow rate of 0.20 m^3^/min. The sampling zone contained the pDR-1500 and tubes to connect the SMPS and APS directly to the chamber, as they operated externally. A honeycomb (AS100, Ruskin, Grandview, MO, USA) flow straightening section divided the two zones and evenly distributed the chamber air for sampling for an aerosol homogeneity of approximately 9% [11]. The chamber contains two 99.9% efficiency HEPA filters to flush it using particle-free air at 1.0 m^3^/min. A vacuum with HEPA and carbon filters removes air from the chamber before and after each experiment. The chamber’s temperature was maintained at 22 ± 1 °C, and the relative humidity was 45 ± 5%, representing controlled indoor laboratory conditions commonly used in aerosol chamber studies. Maintaining stable environmental parameters improves the reproducibility of aerosol mass measurements.

### 2.4. Aerosol Generation

Attached to the chamber was a diaphragm pump (Thomas 1420-0504, Gardner Denver, Davidson, NC, USA) and a clock generator (TFIS 12-240VUC 1CO CG, Weidmüller Interface GmbH & Co. KG, Detmold, Germany) set to “puff” the ECIG device at a flow rate of 2.0 L/min. The clock generator was set to an ECIG puffing time of 3 s and a rest time of 27 s, similar to ECIG users [35]. Each session produced 60 puffs, which is the mean value of two people vaping simultaneously inside their vehicle, as shown in previous work [28]. Each experiment was repeated three times to achieve statistical significance.

### 2.5. Aerosol Monitors

#### 2.5.1. Scanning Mobility Particle Size Spectrometer (SMPS)

The Scanning Mobility Particle Size Spectrometer (SMPS 3938, TSI, Shoreview, MN, USA) system uses a 3082 Classifier, 3081 Long Differential Mobility Analyzer (DMA), 3756 Ultrafine Condensation Particle Counter (CPC), and 3088 Advanced Aerosol Neutralizer to record real-time particle measurements. The SMPS uses 191 bins to separate particles by size from 0.001 µm to 1.0 µm. This system is the standard for sub-micrometer particle measurements at concentrations up to 107 particles per cm^3^, and it classifies particles as spherical when creating PM mass concentrations [12]. For this study, the aerosol flow rate was 0.3 L/min, the sheath air flow rate was 2.0 L/min, and the sampling frequency was 1 min.

#### 2.5.2. Aerodynamic Particle Sizer (APS)

The Aerodynamic Particle Sizer (APS 3321, TSI, Shoreview, MN, USA) spectrometer measures particles from 0.5 to 20 µm using 52 channels with a maximum of 10,000 particles per cm^3^. The APS sizes particles using the Time-Of-Flight, which is needed for particles to travel across a set distance, and it converts the real-time measurements to mass concentrations using the assumed aerosol density [12]. The APS was selected to account for limitations in the SMPS particle size range, and its flow rate was 5.0 L/min with a sampling frequency of 1 min. The APS data complements SMPS data, which are combined to cover the PM_2.5_ range.

#### 2.5.3. Personal DataRAM (pDR-1500)

The personal DataRAM (pDR-1500, Thermo Scientific, Franklin, MA, USA) photometer measures particles in real-time using a cyclone and a set flow rate. The pDR-1500 measures only one particle size and has a maximum concentration of 400,000 μg/m^3^. For this study, the pDR-1500 was set to measure PM_2.5_ with a flow rate of 1.52 L/min and a sampling frequency of 1 s. The pDR-1500 also utilizes a 37 mm filter to collect discrete particle measurements, enabling the calculation of filter correction factors.

#### 2.5.4. Gravimetric Analysis

Fiberglass filters (Whatman, CAT Non. 1827-037, Maidstone, UK) used for pDR-1500 measurements were weighed before and after each experiment to calculate the sample’s mass. Filters were weighed using a Mettler Toledo microbalance (Model: XPR26DR, Colombus, OH, USA) and an anti-static kit with a U-electrode (Model: 63052302, Mettler Toledo, Columbus, OH, USA). The filter equilibration temperature was 22 ± 1 °C and humidity was 45 ± 5% [36]. Discrete filter concentrations for each particle size were calculated by dividing the mass difference by the pDR-1500 flow rate and sampling time for each experiment.

### 2.6. Data Analysis

#### 2.6.1. Nicotine Concentration Differences Between Labeled and Chemical Analysis

Nicotine labeling accuracy was evaluated by calculating the difference between the chemically measured nicotine concentration and the labeled nicotine concentration for each product. Differences were expressed in mg/mL:

Nicotine Difference = Measured Nicotine − Labeled Nicotine(1)

These deviations were visualized as concentration differences across all product groups (N1–N4; 0, 9, 18, and 48 mg/mL) and PG/VG categories (R1–R5).

To determine whether the mean deviation within each nicotine group significantly differed from zero (representing perfect labeling agreement), one-sample t-tests were conducted separately for each labeled nicotine concentration (0, 9, 18, and 48 mg/mL). The test value was set to 0. Each group included five observations (df = 4). Two-sided significance tests were used, with statistical significance defined as *p* < 0.05. Mean differences and 95% confidence intervals were calculated.

#### 2.6.2. PG/VG Ratio Differences Between Labeled and Chemical Analysis

PG/VG ratio labeling accuracy was evaluated by calculating the difference between the chemically measured PG/VG ratio and the labeled PG.VG ratio for each product:

PG/VG Difference = Measured PG/VG Ratio − Labeled PG/VG Ratio(2)

These deviations were visualized as differences in the PG/VG ratio across all labeled PG categories (0%, 30%, 50%, 70%, and 80%) and nicotine groups (N1–N4).

To evaluate whether the mean PG/VG ratio deviation within each labeled PG group significantly differed from zero, one-sample t-tests were conducted separately for each labeled PG percentage category. The test value was set to 0. Each group included four observations (df = 3). Two-sided significance testing was applied with α = 0.05. Mean differences and 95% confidence intervals were calculated.

#### 2.6.3. Real-Time Mass Concentrations

Real-time PM_2.5_ concentrations for the pDR-1500, SMPS, and APS were averaged to 1 min samples and displayed as box-and-whisker plots. The SMPS and APS averages were calculated and combined using a MATLAB (R2023b) code. Box-and-whisker plots were developed to show differences among the ECIG liquid samples used to generate PM_2.5_ emissions.

#### 2.6.4. Filter Correction Factors

Filter correction factor calculations for each flavor and condition combination involved dividing their discrete filter mass concentration by their average real-time PM_2.5_ mass concentration [11]. The filter correction factors enable a “course correction” by comparing real-time device measurements to discrete filter masses.

## 3. Results

### 3.1. Differences in Nicotine Concentration

**Figure 2:**
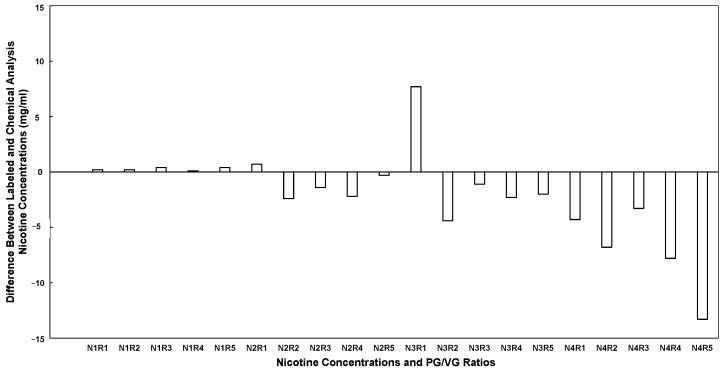
Nicotine concentration differences between ECIG liquid package labels and chemical analysis. Each column represents one value of the difference between measured and labeled values, calculated using Equation (1). Samples are coded according to four nicotine concentrations (N1–N4: 0, 9, 18, and 48 mg/mL) and five PG/VG ratios (R1–R5: 0, 30, 50, 70, and 80). For example, a sample with 0 mg/mL nicotine and a PG/VG ratio of 0 is designated as N1R1, while a sample with 48 mg/mL nicotine and a PG/VG ratio of 80 is designated as N4R5.

The nicotine, PG, and VG concentrations for different products, based on packaging labels and chemical analysis, are presented in Table 1 and Table 2, respectively. In addition, the nicotine concentration differences between the commercial label and chemical analysis for different nicotine and PG/VG ratio samples are shown in Figure 2. The figure presents deviations in nicotine concentration (mg/mL) between labeled and chemically measured values. These deviations ranged from −13.3 mg/mL (N4R5) to +7.7 mg/mL (N3R1). Positive values indicate measured nicotine concentrations exceeding labeled amounts, whereas negative values indicate underfilled products relative to labeling. Positive values indicate measured concentrations exceeding the labeled amount, whereas negative values indicate concentrations below the labeled amount. The chemical analysis revealed discrepancies in nicotine concentration compared to the commercial label across products and ratios. The N1 samples labeled 0 mg/mL contained 0.1–0.4 mg/mL nicotine, demonstrating trace levels despite “nicotine-free” labeling. The N2 samples labeled at 9 mg/mL ranged from 6.6 to 9.7 mg/mL, showing considerable variability, with most samples measuring below the labeled concentration. The N3 samples labeled 18 mg/mL ranged from 13.6 to 25.7 mg/mL. Finally, the N4 samples labeled at 48 mg/mL measured 34.7–44.7 mg/mL, which was significantly lower than the labeled value. Across all products, 35% of samples measured higher nicotine than labeled, and all nicotine-free products contained trace amounts of nicotine. Nicotine concentrations tended to be highest in the R1 formulations with the highest VG content. On average, measured nicotine levels were lower than labeled for the higher-concentration groups (N2, N3, N4), with the greatest underestimation observed in N4 (mean 40.9 mg/mL vs. labeled 48 mg/mL). The nicotine-free N1 products contained small amounts of nicotine (mean 0.26 mg/mL), and variability increased with increasing labeled concentration.

Based on the *t*-test analysis, the mean nicotine difference for the 0 mg/mL products was +0.26 mg/mL, a statistically significant deviation from zero (95% CI: 0.093 to 0.427, *p* = 0.012). Differences were not statistically significant for the 9 mg/mL group (−1.12 mg/mL; 95% CI: −2.75 to 0.51, *p* = 0.129) or the 18 mg/mL group (−0.42 mg/mL; 95% CI: −6.25 to 5.41, *p* = 0.851). In contrast, the 48 mg/mL group showed a significant mean difference of −7.10 mg/mL (95% CI: −11.96 to −2.24, *p* = 0.015).

### 3.2. Differences in PG/VG Ratios

**Figure 3:**
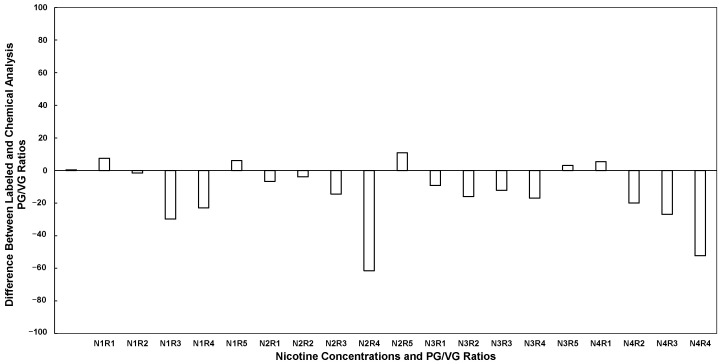
PG/VG ratio differences between the commercial label chemical and chemical analysis for different nicotine and PG/VG ratio samples. Each column represents one value of the difference between measured and labeled values, calculated using Equation (2). Samples are coded according to four nicotine concentrations (N1–N4: 0, 9, 18, and 48 mg/mL) and five PG/VG ratios (R1–R5: 0, 30, 50, 70, and 80). For example, a sample with 0 mg/mL nicotine and a PG/VG ratio of 0 is designated as N1R1, while a sample with 48 mg/mL nicotine and a PG/VG ratio of 80 is designated as N4R5.

The PG/VG ratios and ratio differences between the commercial label chemical and chemical analysis for different nicotine and PG/VG ratio samples were calculated from Table 1 and Table 2, respectively, and are shown in Figure 3. In contrast to nicotine deviations, Figure 3 presents PG/VG ratio differences expressed as percentage-point deviations between labeled and chemically measured solvent composition. These deviations ranged from −61.5 percentage points (N2R5) to +10.8 percentage points (N3R1). Negative values indicate higher VG content than labeled, whereas positive values indicate higher PG content than labeled. Positive values refer to higher PG values, and negative values refer to higher VG values than labeled. The positive values in the figure represent higher PG concentration than expected, compared to negative values that represent higher VG concentrations than labeled. The labeled PG/VG ratios (0/100, 30/70, 50/50, 70/30, 80/20) deviated substantially from chemically measured compositions. A total of 30% of the ratios had positive values, indicating higher PG concentrations, and 70% of the ratios had negative values, indicating higher VG concentrations. Generally, a product labeled as 50/50 is often tested closer to 46/54 or even 34/66. Conversely, a 100% VG product contained measurable PG concentrations, as chemical analysis in all R1 cases (N1R1, N2R1, N3R1, N4R1) revealed that the products contained up to ~10% PG. The densities ranged from 1.08 to 1.26 g/mL, depending on the PG/VG ratio. Measured densities were slightly different, generally within 0.01 to 0.05 g/mL of the labeled ratios. However, density variations were smaller compared to discrepancies in the PG/VG ratios.

Based on the t-test analysis, the mean deviation for products labeled 0% PG (100% VG) was +5.10 percentage points, which was not statistically significant (95% CI: −1.99 to 12.19, *p* = 0.106). Deviations were also not statistically significant for the 30% PG group (−0.75 percentage points; 95% CI: −14.15 to 12.65, *p* = 0.870) or the 50% PG group (−10.33 percentage points; 95% CI: −24.65 to 3.99, *p* = 0.106). In contrast, significant mean deviations were observed for the 70% PG group (−20.80 percentage points; 95% CI: −34.88 to −6.72, *p* = 0.018) and the 80% PG group (−38.43 percentage points; 95% CI: −73.11 to −3.74, *p* = 0.039).

### 3.3. Discrepancies in Real-Time Mass Concentrations

**Figure 4:**
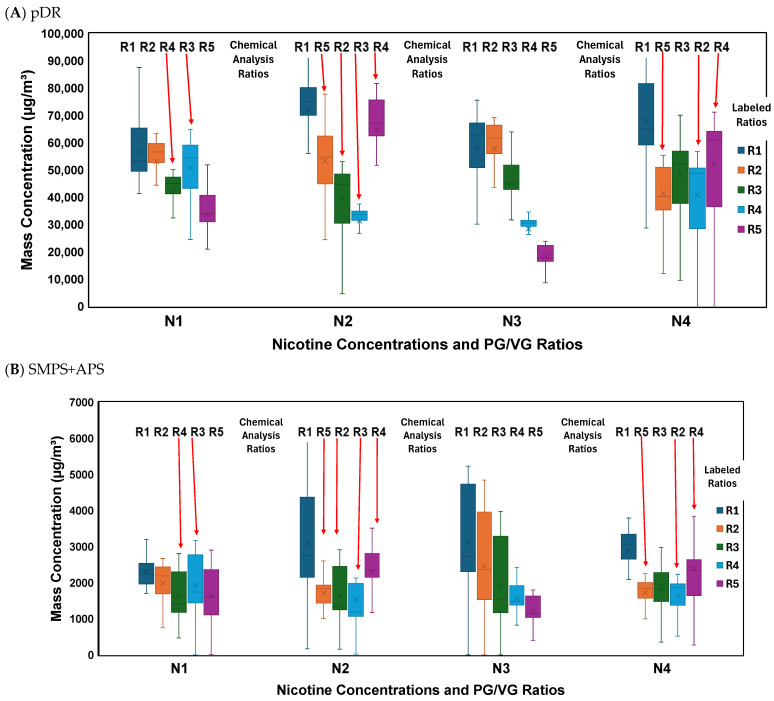
Box and whisker plots (**A**) pDR and (**B**) SMPS+APS real-time raw PM_2.5_ mass concentration measurements averaged over three trials. Samples are coded according to four nicotine concentrations (N1–N4: 0, 9, 18, and 48 mg/mL) and five PG/VG ratios (R1–R5: 0, 30, 50, 70, and 80). For example, a sample with 0 mg/mL nicotine and a PG/VG ratio of 0 is designated as N1R1, while a sample with 48 mg/mL nicotine and a PG/VG ratio of 80 is designated as N4R5. The Box and Whisker columns represent the “labeled ratios” as identified by the legend, and the R1–R5 labels on top of the Box and Whisker columns represent the “Chemical Analysis” ratios.

The box-and-whisker plots of the pDR and SMPS+APS raw (not filter-corrected) real-time PM_2.5_ mass concentration measurements, averaged over three trials per liquid, are shown in Figure 4. Nicotine concentrations were designated as N1 through N4 in the figures, and PG/VG ratios were ordered based on labeled values from R1 (left) to R5 (right). Chemically verified PG/VG ratios were overlaid on the box-and-whisker plots, with arrows highlighting discrepancies between labeled compositions and chemical analysis results. Based on solvent composition, PM_2.5_ concentrations are expected to decrease as VG content decreases, with the highest concentrations on the left (highest VG) and the lowest at the right (lowest VG). However, this expected trend is obscured when samples are ordered by labeled PG/VG ratios due to mislabeling. For example, for the pDR measurements at N1, the labeled R3 condition (ratio) shows lower concentrations than the labeled R4 condition, because the actual values are reversed based on the chemical analysis results. When samples are reordered according to chemically verified PG/VG ratios (overlaid ratios), the expected downward trend in PM_2.5_ concentration becomes evident.

For both the pDR and SMP+APS, the mass concentrations were generally higher from left to right, starting with R1 at the highest VG, and ending with the corrected measured ratio. PM_2.5_ concentrations were slightly higher for N4 (higher nicotine) than N1 across most ratios. However, the difference was modest, less than ~7% for the pDR and lower for the SMPS+APS. This may be due to nicotine influencing aerosol formation.

### 3.4. Discrepancies in Filter Correction Factors

The filter correction factor (FCF) and FCF standard deviation (SD) values in chemical analysis results per nicotine concentrations and PG/VG percentages are shown in Table 3. The FCF values can be used to correct the raw PM_2.5_ concentrations in Figure 4. For the pDR, the FCF values were remarkably stable across all samples. Its value remains consistently low, averaging around 0.42 and ranging between 0.39 and 0.48. The standard deviation (FCF SD) was also very small (0.01–0.03), indicating high measurement consistency. The change in nicotine concentration had no tangible effect; samples from nicotine-free (N1) through high-nicotine (N4) remained ~0.4–0.46. The PG/VG ratios effects showed a slight upward trend with higher PG (e.g., N1R1 at 0.39 with ~0.4% PG vs. N1R5 at 0.46 with 57% PG). The results showed that the pDR systematically overestimates PM_2.5_ by about 42% compared to the filter, regardless of ECIG liquid composition, but PG-rich samples tend to nudge the FCF slightly higher. For the SMPS+APS, the FCF values were high and variable, averaging approximately 11.2 and ranging from 6.88 to 12.62. When comparing the two devices, pDR vs. SMPS+APS, the SMPS+APS’s FCF values (around 11) were consistently 25 to 30 times higher than the pDR’s FCF values (around 0.4). The pDR’s FCF values were extremely stable with a low standard deviation. The SMPS+APS’s FCF values were much more variable, with a standard deviation (FCF SD) often 100 times larger than the pDR’s.

## 4. Discussion

The study used an ECIG device to generate aerosols from unflavored liquids purchased from a single retailer to minimize variability. Liquids were purchased at four nicotine concentrations and five PG/VG ratios, and chemical analysis focused on nicotine, PG, and VG content. Aerosol emissions were produced inside a controlled exposure chamber by a diaphragm pump and a clock generator operating. Particle measurements were collected using an SMPS, an APS, and a pDR-1500 photometer to measure real-time PM_2.5_ mass. Gravimetric analysis was performed to derive filter correction factors. Discrepancies were evaluated between the labeled and measured chemical content and their effects on real-time and discrete measurements. Figure 2 and Figure 3 are plotted across the same sample identifiers (N1R1–N4R5); they represent independent deviation metrics with different units and substantially different magnitudes, reflecting separate labeling discrepancies in nicotine concentration and solvent composition. It is important to distinguish between deviations in nicotine concentration and deviations in the PG/VG solvent ratio. While both were plotted across identical sample identifiers, nicotine discrepancies were generally within ±13 mg/mL, whereas PG/VG discrepancies reached as high as −61.5 percentage points. The magnitude of solvent mislabeling was therefore substantially greater than that of nicotine mislabeling, with direct implications for aerosol mass formation and instrument response.

The chemical analysis demonstrated notable inconsistencies between labeled and measured nicotine concentrations across ECIG liquids, suggesting variability in manufacturing accuracy and quality control. Although the trace nicotine detected in “nicotine-free” products (N1) was low, its presence raises concerns for users intentionally seeking nicotine-free options, including youth, individuals attempting cessation, and those sensitive to nicotine exposure. Variability increased with higher labeled concentrations. Underfilled high-strength nicotine liquids may alter user exposure, limit satisfaction, and again encourage compensatory use patterns. Studies consistently report similar discrepancies in commercial ECIG liquid, with actual nicotine levels often deviating from labels. Kim, et al. [37] conducted a study of 32 electronic cigarette liquid refill products purchased in 2014 in South Korea. The study revealed significant labeling inconsistencies, with nicotine concentration discrepancies ranging from −32.2% to 3.3% compared to labeled values. The actual nicotine levels varied widely, from undetectable to 17.5 mg/mL, with one “pure nicotine” product with no concentration on the label, yielding a dangerously high 150.3 mg/mL measured value. The lack of standardized labeling and the presence of potentially hazardous products highlight the urgent need for regulatory oversight. Goniewicz, et al. [38] conducted a study of 16 popular electronic cigarette (EC) brands from Poland, the UK, and the US, and found that the nicotine content revealed that 9 out of 20 cartridges and 3 out of 15 refill solutions had nicotine levels differing by more than 20% from manufacturer-declared values, indicating significant labeling inconsistencies for some brands. However, most values were lower, consistent with this study, in which most nicotine values were also lower than the label indicated. The one-sample *t*-test results demonstrate that nicotine mislabeling was concentration-dependent. Products labeled as nicotine-free contained small but statistically significant amounts of nicotine, confirming contamination or incomplete nicotine removal during manufacturing. At the opposite end of the concentration spectrum, the 48 mg/mL group exhibited significant under-labeling, with measured nicotine concentrations averaging 7.1 mg/mL and a maximum of 13.3 mg/mL below labeled values. In contrast, the intermediate concentrations (9 and 18 mg/mL) did not significantly deviate from labeled values. This suggests that labeling inaccuracies were not uniform across nicotine strengths. The significant deviation at 0 mg/mL and 48 mg/mL indicates potential formulation or quality control inconsistencies at the lowest and highest production levels.

Chemical analysis of the PG/VG ratios revealed deviations from the labeled compositions, indicating inconsistencies in the formulation of ECIG liquids across nicotine concentrations and ratio categories. Most samples (70 percent) contained higher VG levels than labeled, while only 30 percent contained higher PG levels, demonstrating a consistent trend toward VG-rich mixtures. These deviations were not minor. Products labeled as 50/50 were commonly measured closer to 46/54 or even 34/66, changes large enough to influence aerosol production, throat hit, and overall device performance. Detectable levels of PG were found in the 100% VG product, with chemical profiling across the R1 cases (N1R1 through N4R1) indicating a composition of up to ~10% PG. The detection of measurable propylene glycol (PG) in products labeled as 100% vegetable glycerin (VG) may be related to formulation practices, as extracted nicotine is typically prepared in VG/PG [39]. Alternative causes for unexpected PG levels stem from the fact that e-cigarette formulations are ‘virtually unregulated’ [40]. Because of this, it is not unexpected to find ‘glycol concentrations different from the labeled concentrations’ [40], with studies frequently documenting ‘mislabeled’ products and significant discrepancies in stated versus actual propylene glycol to glycerin ratios [40,41]. Additionally, compositional discrepancies and difficulties in accurate quantification can stem from imperfect mixing and incomplete homogenization, particularly due to the high viscosity of vegetable glycerin [42]. 

Regarding purity, density values, which depend on PG/VG ratios, also showed discrepancies, leading to deviated density calculations. Chemical analysis reveals significant and widespread inaccuracies between the labeled and actual PG/VG ratios in the tested samples. The discrepancies range from minor deviations to complete inversions of the advertised content. No prior work has examined discrepancies in PG/VG ratio and density calculations. The results of this work underscore that higher VG content is typically available than advertised, and although this reduces nicotine absorption relative to high-PG products, it may leave consumers craving more puffs to satisfy their nicotine need [17]. Increasing VG content can also cause short-term throat irritation, dry cough, and temporary reductions in lung function at high exposure, which could occur due to increased intake to offset nicotine needs [18]. Unlike nicotine concentration deviations, PG/VG ratio discrepancies were more pronounced at higher labeled PG values. While the 0%, 30%, and 50% PG groups did not show statistically significant differences from zero, the 70% and 80% PG groups demonstrated significant negative deviations. This indicates that products labeled with high PG content consistently contained substantially lower PG percentages than stated. The magnitude of solvent mislabeling was considerably greater than that of nicotine mislabeling. For example, the 80% PG group exhibited a mean deviation of −38.4 percentage points, whereas the largest nicotine deviation was −13.3 mg/mL in the 48 mg/mL group. These results suggest that solvent composition inaccuracies may represent a more substantial quality control issue than nicotine concentration inaccuracies.

From a mechanistic perspective, the solvent-driven changes in PM_2.5_ emissions align with established aerosol thermodynamics. Vegetable glycerin (VG), which has a lower vapor pressure and higher boiling point than propylene glycol (PG), promotes formation of larger and more persistent droplets, resulting in higher measured particulate mass concentrations [11,12]. In contrast, PG-rich aerosols are more volatile and readily evaporate, particularly in dilution-based instruments such as the SMPS, where sheath air enhances particle shrinkage and mass underestimation [12]. This volatility-driven behavior explains the elevated and variable correction factors observed for the SMPS+APS system compared with the more stable pDR response. Practically, solvent mislabeling has important implications for exposure assessment and regulatory evaluation. Because PM_2.5_ formation is strongly governed by solvent composition rather than nicotine concentration, inaccurate PG/VG labeling may bias comparative emission studies and risk characterization. Routine chemical verification of liquid composition may therefore improve reproducibility and strengthen quality-control oversight [37,38].

Mass concentrations measured by both the pDR and SMP+APS generally showed an upward trend, progressing from the high-VG R1 samples to the corrected measured ratios. For both the pDR and SMP+APS, the mass concentrations were generally higher from left to right, starting with R1 at the highest VG, and ending with the corrected measured ratio. These findings align with the results of Sousan et al. [11], where PM_2.5_ concentrations were highest at high VG content and lowest with high PG content, due to the low vapor pressure and high volatilization of the particles, which caused them to evaporate into the vapor phase. The pDR PM_2.5_ concentrations were consistently much higher than SMPS+APS PM_2.5_ concentrations. This was similar to Close et al. [12] finding, where both the SMPS and the APS experience PG losses due to additional evaporation from the sheath air added to dilute the inlet sample, so the particle count remains detectable within the device specifications. These results confirm that the labels were incorrect, likely due to poor quality control, as the PM_2.5_ mass concentrations were consistent with the actual measured values for the chemical analysis.

The FCFs derived from the SMPS+APS were both high and variable, and these values indicate that the SMPS+APS underestimated the true values of the PM_2.5_ mass concentrations by 1100% due to the volatilization when mixed with sheath air, which aligns with Figure 4 results [12]. The effect of nicotine on FCFs was weak, with values generally increasing from nicotine-free liquids (N1) to mid-level concentrations (N2, N3) and stabilizing at the highest nicotine level (N4). In contrast, PG/VG ratios showed a clearer pattern: higher VG content produced higher FCFs, while PG-rich mixtures yielded the lowest corrections. For instance, high VG samples (~90%) produced FCFs of 9–10, whereas a high PG sample (63%) had the lowest FCF at 6.88, suggesting that PG-rich aerosols evaporate more readily and require a smaller correction. The SMPS+APS produced FCF values were not only 25–30 times higher than the pDR but also significantly more variable. While the pDR demonstrated exceptional stability with low standard deviations. These results were consistent with those of Close et al. [12], where the pDR FCF values ranged from 0.5 to 1.07, compared to 4.96 and 7.62 for the SMPS+APS. The variation in FCF values between this study and Close et al. [12] was primarily due to the different ECIG liquids and atomizers used.

This study was not designed to conduct a quantitative health risk assessment; the findings have implications for hazard interpretation. Chamber PM_2.5_ concentrations reflect controlled-emission conditions and should not be interpreted as real-world bystander doses without accounting for ventilation, room volume, proximity, and behavioral factors. Nonetheless, the directionality of the results is relevant because PM_2.5_ is consistently linked with adverse cardiovascular outcomes across short- and long-term exposure windows in large syntheses and regulatory reviews [43,44]. In addition, labeling discrepancies may be more significant for vulnerable groups. Adolescents are uniquely susceptible to nicotine dependence and neurodevelopmental effects, and authoritative reviews caution that nicotine can harm the developing brain [45]. For pregnancy, public health guidance indicates nicotine exposure is unsafe for the pregnant person and fetus and can adversely affect fetal development [46,47]. Accordingly, mislabeling of “nicotine-free” or solvent composition may create unrecognized exposure potential and complicate product risk communication and regulatory evaluation [48].

**Study Limitations**

This study does not directly assess the health implications of e-liquid labeling discrepancies for passive bystanders. In real-world indoor settings, exhaled aerosols rapidly dilute and disperse, meaning secondhand exposure is driven more by ventilation and room volume than by modest deviations between labeled and actual e-liquid composition. As a result, the toxicological relevance of labeling discrepancies for bystanders may be limited compared with their direct impact on users.

Methodologically, this study assessed nicotine and PG/VG concentrations using a single online e-cigarette retailer. While this reduced cross-vendor variability, it limits the extrapolation of these findings to the broader ECIG marketplace. The discrepancies observed may reflect retailer-specific practices rather than industry-wide patterns driven by variations in production standards, ingredient sourcing, storage, or distribution channels. Furthermore, because this study focused on chemical verification rather than manufacturing process analysis, and did not include replicate bottles from different batches, the data cannot distinguish systematic formulation bias from batch-to-batch variability. Therefore, the magnitude and direction of mislabeling reported in this study should not be interpreted as representative of all ECIG products sold by different manufacturers.

To identify the underlying sources of PG/VG deviations and better assess manufacturing reproducibility, future investigations should include multiple retailers, multiple manufacturers, brands, replicate batches, and manufacturer-supplied formulation data, to distinguish systematic labeling bias from batch-to-batch variability. Additionally, this study relied solely on the SMPS, APS, and pDR-1500, whereas similar chamber studies have employed supplementary reference monitors to derive more comprehensive filter correction factors [11,12]. Finally, previous work has demonstrated that flavorant chemical structures change upon vaporization, which may affect PM_2.5_ mass concentrations [49].

## 5. Conclusions

This study demonstrates that commercially available e-cigarette liquids obtained from a single retailer exhibit substantial discrepancies between labeled and chemically verified nicotine concentrations and PG/VG ratios. By pairing NMR-based chemical analysis with controlled chamber measurements, the results show that mislabeling of liquid composition can alter expected PM_2.5_ emission trends and influence real-time instrument response and gravimetric correction behavior. Importantly, these findings highlight how reliance on labeled liquid properties, rather than verified composition, can lead to mischaracterization of aerosol emissions under laboratory conditions. While inaccuracies in nicotine concentration and PG/VG ratios are most directly relevant to users who inhale the aerosol, the present study was not designed to quantify realistic indoor exposure or passive bystander dose, which depend strongly on environmental dilution, ventilation, and spatial dispersion.

Discrepancies in PG/VG composition may have practical implications for users in real-world settings, where lower-than-expected PG content can reduce nicotine delivery efficiency and potentially encourage compensatory puffing behavior. Conversely, higher-than-labeled VG content can increase aerosol density, which may alter user sensory experience and increase irritation relative to what is anticipated based on product labeling.

Overall, the results emphasize the importance of chemical verification when interpreting aerosol emission measurements from ECIG liquids. Future work should extend this approach across multiple manufacturers, formulations, and device types, and incorporate larger environmental volumes to better link source-term variability to real-world exposure scenarios.

## Figures and Tables

**Table 1:** Nicotine, PG, and VG concentrations for different products based on commercial labels.

Name	Nic. Conc. (mg mL^−1^)	Nic. Conc. (% per 100 mL)	PG	VG	PG/VG Density
N1R1	0.0	0.00	0	100	1.26
N1R2	0.0	0.00	30	70	1.19
N1R3	0.0	0.00	50	50	1.15
N1R4	0.0	0.00	70	30	1.11
N1R5	0.0	0.00	80	20	1.08
N2R1	9.0	0.90	0	100	1.26
N2R2	9.0	0.90	30	70	1.19
N2R3	9.0	0.90	50	50	1.15
N2R4	9.0	0.90	70	30	1.11
N2R5	9.0	0.90	80	20	1.08
N3R1	18.0	1.80	0	100	1.26
N3R2	18.0	1.80	30	70	1.19
N3R3	18.0	1.80	50	50	1.15
N3R4	18.0	1.80	70	30	1.11
N3R5	18.0	1.80	80	20	1.08
N4R1	48.0	4.80	0	100	1.26
N4R2	48.0	4.80	30	70	1.19
N4R3	48.0	4.80	50	50	1.15
N4R4	48.0	4.80	70	30	1.11
N4R5	48.0	4.80	80	20	1.08

**Table 2:** Nicotine, PG, and VG concentrations for different products based on chemical analysis.

Name	Nic. Conc. (mg mL^−1^)	Nic. Conc. (% per 100 mL)	PG	VG	PG/VG Density
N1R1	0.2	0.02	0.4	99.6	1.26
N1R2	0.2	0.02	37.5	62.5	1.18
N1R3	0.4	0.04	48.5	51.5	1.17
N1R4	0.1	0.01	40.2	59.8	1.15
N1R5	0.4	0.04	57.1	42.9	1.13
N2R1	9.7	0.97	6.1	93.9	1.25
N2R2	6.6	0.66	23.3	76.8	1.22
N2R3	7.6	0.76	46.1	53.9	1.21
N2R4	6.8	0.68	55.6	44.4	1.16
N2R5	8.7	0.87	18.5	81.5	1.14
N3R1	25.7	2.57	10.8	89.2	1.24
N3R2	13.6	1.36	20.8	79.2	1.21
N3R3	16.9	1.69	34.0	66.0	1.19
N3R4	15.7	1.57	57.9	42.1	1.13
N3R5	16.0	1.60	63.0	37.0	1.12
N4R1	43.7	4.37	3.1	96.9	1.25
N4R2	41.2	4.12	35.4	64.6	1.20
N4R3	44.7	4.47	30.1	69.9	1.19
N4R4	40.2	4.02	43.1	56.9	1.18
N4R5	34.7	3.47	27.7	72.3	1.17

**Table 3:** The filter correction factor (FCF), and FCF standard deviation (SD) values in order of chemical analysis results per nicotine concentrations and PG/VG percentages.

Chemical Analysis Values				pDR-1500		SMPS+APS	
Name	Nicotine Conc. (mg/mL)	PG (%)	VG (%)	FCF Mean	FCF SD	FCF Mean	FCF SD
N1R1	0.2	0.4	99.6	0.39	0.01	9.7	0.44
N1R2	0.2	37.5	62.5	0.42	0.01	11.43	1.74
N1R4	0.1	40.2	59.8	0.39	0.01	10.81	1.8
N1R3	0.4	48.5	51.5	0.43	0.03	11.87	1.89
N1R5	0.4	57.1	42.9	0.46	0.01	10.29	2.4
N2R1	9.7	6.1	93.9	0.43	0.01	10.9	2.96
N2R5	8.7	18.5	81.5	0.44	0	12.48	2.26
N2R2	6.6	23.2	76.8	0.41	0.02	12.62	1.52
N2R3	7.6	46.1	53.9	0.42	0.02	10.57	2.28
N2R4	6.8	55.6	44.4	0.46	0.01	10	2.56
N3R1	25.7	10.8	89.2	0.42	0.04	9.15	3.99
N3R2	13.6	20.8	79.2	0.44	0.01	11.67	3.88
N3R3	16.9	34	66	0.42	0	11.63	3.8
N3R4	15.7	57.9	42.1	0.48	0.03	9.06	2.04
N3R5	16	63	37	0.45	0.02	6.88	0.55
N4R1	43.7	3.1	96.9	0.42	0.03	10.08	1.8
N4R5	34.7	27.7	72.3	0.43	0.04	9.53	1.73
N4R3	44.7	30.1	69.9	0.43	0.02	11.28	1.09
N4R2	41.2	35.4	64.6	0.42	0.03	10.03	1.61
N4R4	40.2	43.1	56.9	0.44	0.03	10.84	1.74

## Data Availability

The data presented in this study are available on request from the corresponding author.
